# Novel TSURECOMI Bailout Technique for Transcatheter Heart Valve Implantation After Accidental Wire Withdrawal During TAVR

**DOI:** 10.1016/j.jaccas.2021.06.017

**Published:** 2021-08-19

**Authors:** Akimitsu Tanaka, Masayuki Nakamura, Hidekazu Aoyama, Ryosuke Kametani

**Affiliations:** Department of Cardiology, Nagoya Tokushukai General Hospital, Aichi, Japan

**Keywords:** bailout techniques, INDY, lasso technique, snare, transarterial embolization, LV, left ventricle, TAVR, transcatheter aortic valve replacement, THV, transcatheter heart valve, TSURECOMI, Transarterial Snare-Upholding REcovery technique for COMpletely pulled out LV wire for TAVR valve Insert system

## Abstract

This study introduces a case in which our novel “Transarterial Snare-Upholding REcovery technique for COMpletely pulled out LV wire for TAVR valve Insert system (TSURECOMI) technique” with snares was successfully performed for bailout of a transcatheter heart valve during transcatheter aortic valve replacement. (**Level of Difficulty: Advanced.**)

## Central Illustration

**Figure:**
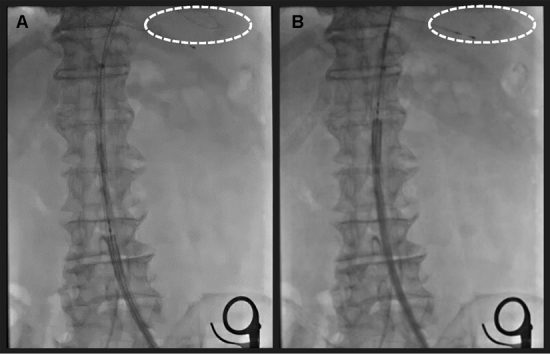

The Sapien transcatheter heart valve (THV) (Edwards Lifesciences) incorporates a simple deployment system and is one of the most frequently used THV devices worldwide. During transcatheter aortic valve replacement (TAVR), a stiff wire is typically inserted into the left ventricle (LV) through the aortic valve and then used to guide the THV to the aortic valve for implantation. However, it is important to note that, if this type of THV is inserted and subsequently removed from the body, it cannot be reused. Because this type of THV is very expensive, accidental withdrawal of the device poses a clinical challenge for the procedure (1).

**Learning Objectives**

• To illustrate our novel TSURECOMI technique for bailout of a THV during TAVR if a guidewire is accidentally withdrawn from the LV.
• To confirm that the TSURECOMI technique is not invasive or expensive and can be safely performed by any interventional cardiologist or cardiac surgeon.

This paper describes a case in which a THV bailout was successfully and safely performed using snares and the authors’ novel Transarterial Snare-Upholding REcovery technique for COMpletely pulled out LV wire for TAVR valve Insert system (TSURECOMI) technique.

## Medical History and History of Presentation

An 82-year-old man with dyspnea on exertion was referred to our hospital. His medical history was unremarkable.

## Differential Diagnosis

Coronary computed tomography revealed that the patient’s coronary arteries were intact, indicating that he had no ischemic heart disease.

## Investigations

The patient had an obvious systolic heart murmur, which suggested severe aortic valve stenosis; therefore, echocardiography was performed. The results confirmed severe stenosis; therefore, the authors decided to treat the patient by performing TAVR. First, a soft and straight Radifocus (Terumo Medical) wire was inserted into the LV and was crossed with a 4-F catheter (AL1). Next, the soft and straight wire was exchanged with a stiff Safari wire (Boston Scientific). Finally, a Sapien 3 THV was inserted through an expandable sheath by mounting it on a Commander transfemoral delivery system (Edwards Lifesciences).

**Figure 1:**
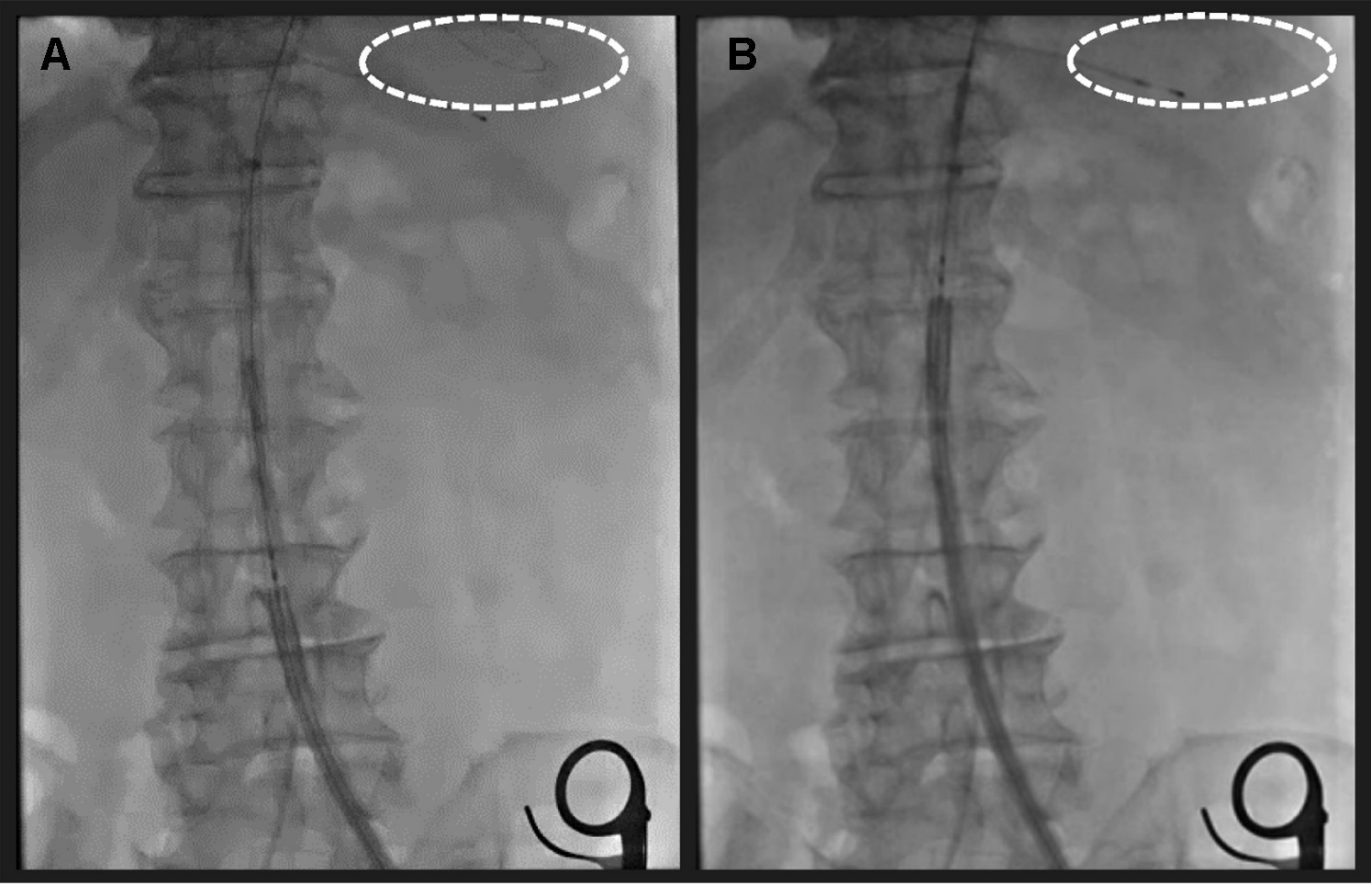
A Stiff Guidewire is Accidentally Withdrawn From the Left Ventricle **(A)** The tip of the wire is inside the left ventricle **(white circle)**. **(B)** The tip of the wire is outside the LV **(white circle)**.

During the procedure, the authors realized that the stiff wire, initially intended for placement in the LV, had accidentally been withdrawn (Figure 1). Typically, when the transfemoral delivery system is inserted, an assistant surgeon holds the stiff wire and prevents it from being drawn into the body. However, in this case, the assistant surgeon apparently pulled on the wire more forcefully than required during the procedure, resulting in accidental withdrawal of the stiff wire from the LV. A THV cannot be inserted into the LV through a severely stenotic aortic valve without a stiff wire. Furthermore, once the THV is withdrawn from the body, it cannot be reused, and the cost to replace it is very high.

## Management

**Figure 2:**
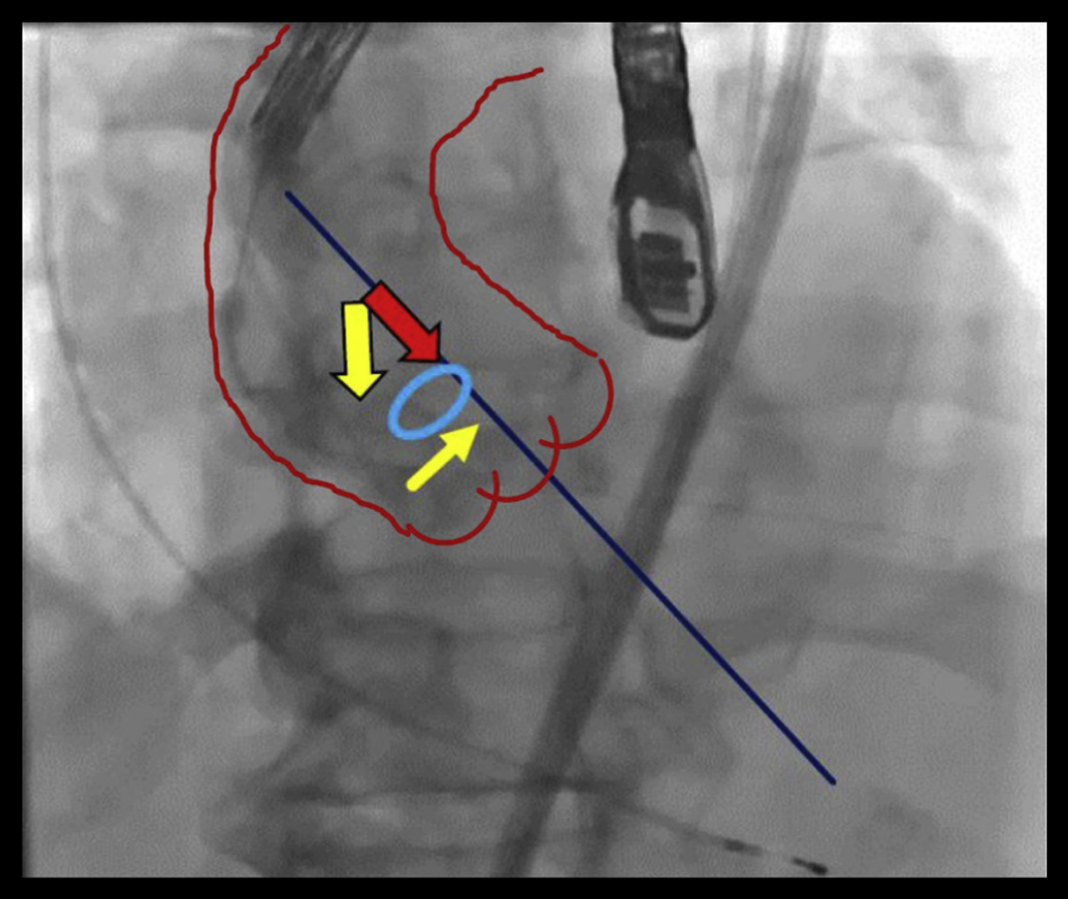
Ineffective Lasso Technique in Transcatheter Aortic Valve Replacement The force vector was directed toward the inner side and not the outer side of the aortic curve using the Lasso technique. The force vector of the downward plus the rightward **yellow arrows** is equal to that of the **red arrow**.

Following its accidental withdrawal from the LV, the stiff wire was withdrawn from the delivery system. Subsequently, the authors crossed a soft and straight wire instead of a stiff wire into the LV through the aortic valve. However, as expected, the wire proved to be too soft to insert even the tip of the delivery system into the LV; therefore, the force vector was directed toward the outer side of the aortic curve. Next, the tip angle of the delivery system was adjusted using its flexible catheter, but this approach was unsuccessful. Hence, the authors attempted to direct the force vector toward the inner and not the outer side of the aortic curve by using the lasso technique with snares (Figure 2) (2).

For this procedure, the authors first exchanged the sheath for a pigtail catheter (which had been inserted into the other femoral site) for a larger 8-F sheath. As described in the beginning of the Investigation section (above), a soft and straight wire was inserted into the LV again using a 4-F catheter (AL1) through the 8-F sheath and then exchanged the soft and straight wire for a stiff wire.

**Figure 3:**
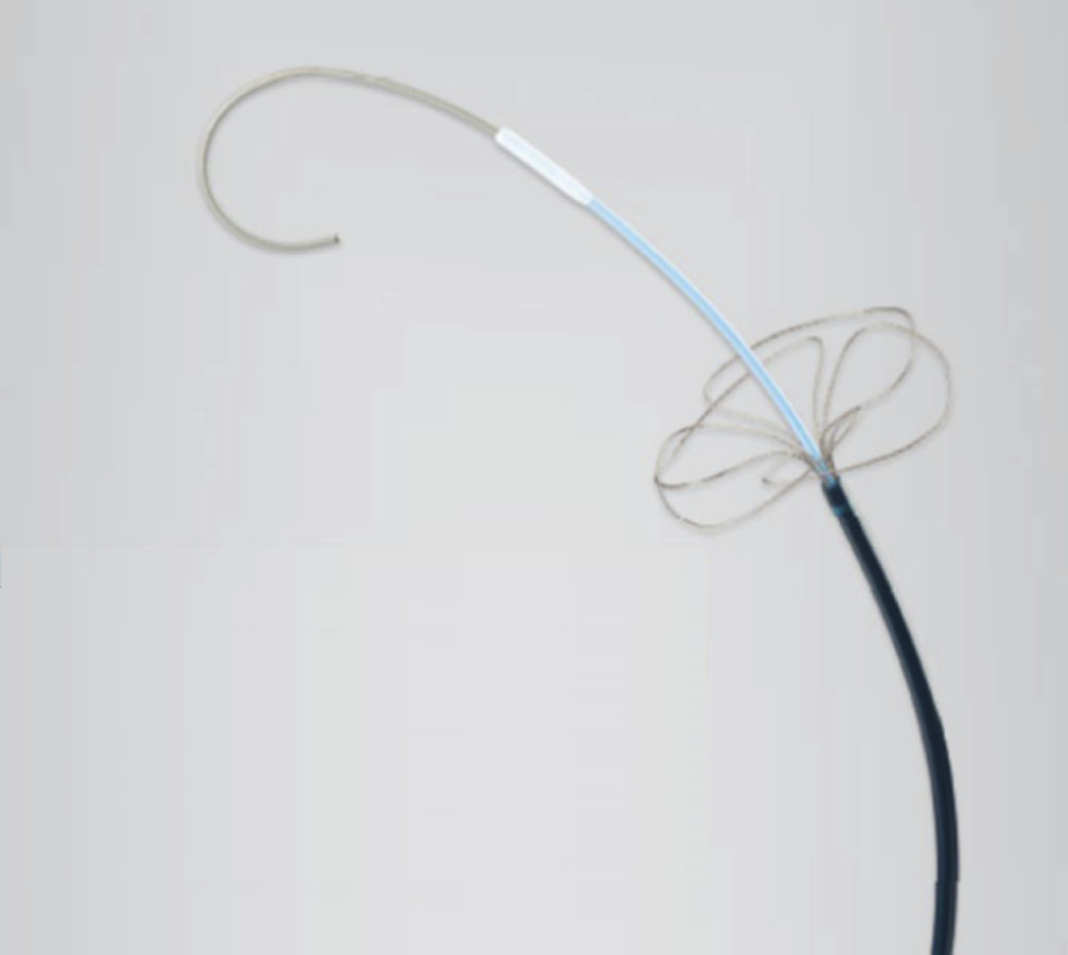
Image of the Over-the-Wire Snare Used in the TSURECOMI Technique The snare is inserted into an 8-F sheath and may be used as a 6-F sheath. TSURECOMI = Transarterial Snare-Upholding REcovery technique for COMpletely pulled out LV wire for TAVR [transcatheter aortic valve replacement] valve Insert system.

Next, an INDY OTW (Cook Medical Japan GK) over-the-wire snare (Figure 3) was inserted by using the stiff wire through the 8-F sheath and caught the delivery system tip with the snare. (Note that we had previously withdrawn the initial wire in the delivery system from the LV because, at that time, we were unable to catch the tip of the delivery system with a snare.) We then crossed the soft and straight wire from the delivery system into the LV and attempted to direct the force vector toward the inner (and not the outer) side of the aortic curve to push the delivery system into the aortic valve. However, this approach was also unsuccessful.

After consideration, it was realized that we should not change the force vector but instead should push the snare directly into the LV with the delivery system. Using this method, it was found that the snare did not support entry of the THV into the LV, but rather it took the THV into the LV.

The type of snare used in this technique is crucial to ensure its success. The most common type of snare can “catch and pull” but cannot “catch and push” a target object. In this case, an over-the-wire snare was used with a “rail” in both the posterior and anterior directions, enabling it to catch and push the THV into the LV.

**Figure 4:**
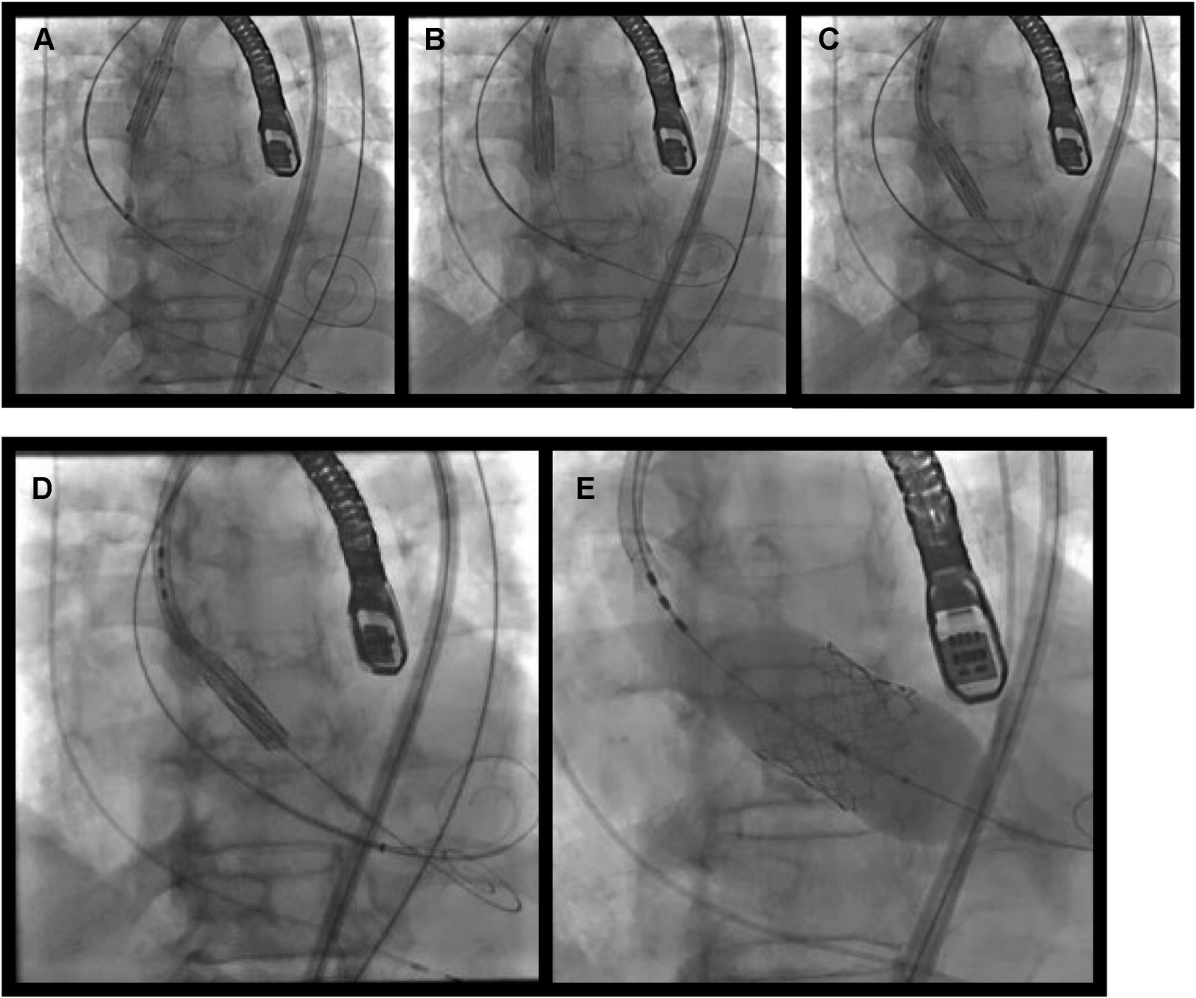
Steps in the Deployment of a Transcatheter Heart Valve **(A to C)** The over-the-wire snare was progressed to the left ventricle by firmly catching the delivery system. **(D and E)** We deployed the transcatheter heart valve to the aortic valve annulus by the usual method.

The over-the-wire snare was progressed to the LV by firmly catching the delivery system and finally succeeded in inserting it into the aortic annulus. We then advanced another stiff wire (which had previously been incorporated in the delivery system) and inserted a pigtail catheter after withdrawing the over-the-wire snare and stiff wire from the patient. In this way, we safely deployed the THV to the aortic valve annulus by the usual method (Figure 4).

We call this novel technique the TSURECOMI technique, which means “*take someone to a place*” in Japanese, and is an acronym for Transarterial Snare-Upholding REcovery technique for COMpletely pulled out LV wire for TAVR valve Insert system.

The following is a step-by-step description of the TSURECOMI technique:

1. Insert an 8-F sheath into the femoral artery that was not used for TAVR.
2. Insert a soft and straight wire into the LV and cross a 4-F catheter (AL1 or JR4, among others) through the 8-F sheath. Next, exchange the soft and straight wire for a stiff J-tip wire.
3. Insert an over-the-wire snare over the stiff wire through the 8-F sheath.
4. Catch the tip of the delivery system with the snare.
5. Advance the snare to the LV while firmly attached to the delivery system.

**Figure 5:**
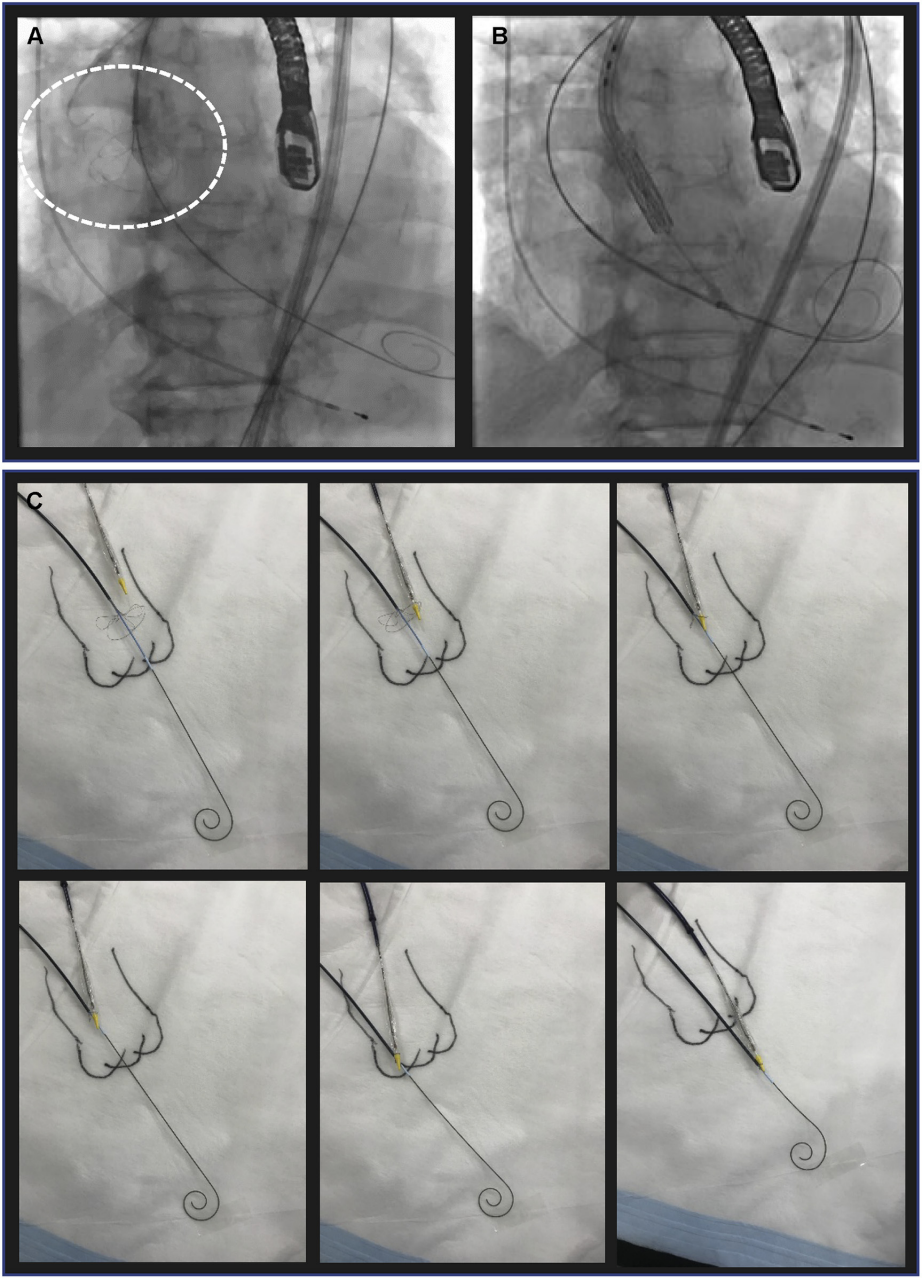
Outline of the TSURECOMI Technique **(A)** Another stiff J-tip wire was inserted into the left ventricle, and an over-the-wire snare on the stiff wire was inserted to catch the tip of the transfemoral delivery system **(white circle)**. **(B)** The over-the-wire snare was advanced to the left ventricle while firmly attached to the delivery system. In this way, the delivery system is TSURECOMareru (the delivery system is automatically “*taken*” into the left ventricle). **(C)** The TSURECOMI schema.

When this technique is used, the snare seems as if it catches and takes the delivery system to the LV (Figure 5, Video 1).

## Discussion

If a guidewire is accidentally withdrawn from the LV while inserting a THV, it may be possible to recross a straight-tip wire through the aortic valve by taking advantage of the flexibility of the tip of the delivery system. If the tip of the device can be introduced through the aortic valve, then a stiff J-tip wire can often be successfully positioned in the LV, enabling full crossing with the THV and subsequent deployment. However, this approach is not always successful. The use of a hard-type wire with a straight tip and advancement of a delivery system over the stiff wire is one of the alternatives in these situations, but it may incur a risk of LV injury with a possibility of cardiac tamponade.

As one of the methods that can be performed in such situations, it may be possible to catch the wire with a snare using the transapical approach or the transseptal approach using the Brockenbrough method. However, both techniques are invasive and require significant experience. Withdrawing the THV from the body with an expandable sheath and then inserting a new one is the simplest approach to overcome this problem. Edwards Lifesciences states that the Sapien 3 valve can be withdrawn into the expandable sheath and then removed. Nevertheless, this is not always possible in practice, and the potential for sticking or dislodgement of the THV or vascular injury to the femoral vessels is significant. In addition, the cost of another THV is extremely high.

We resolved this challenge by using the TSURECOMI technique with an over-the-wire snare. This technique is neither invasive nor expensive. Furthermore, it can be safely and easily performed by any interventional cardiologist or cardiac surgeon without extensive experience. Unlike the lasso technique, this method does not require a difficult wiring procedure over the transfemoral delivery system.

Furthermore, this technique is potentially useful in cases requiring deployment of a second valve to replace a self-expandable TAVR valve that has accidentally popped up, or if a deteriorated TAVR valve must be replaced with a new one.

## Follow-Up

The patient was asymptomatic at the 1-year follow-up visit.

## Conclusions

Our TSURECOMI technique is very useful for bailout of the most commonly used THV during TAVR in cases where a guidewire is accidentally withdrawn from the LV.

## Funding Support and Author Disclosures

The authors have reported that they have no relationships relevant to the contents of this paper to disclose.

## Appendix

Supplemental Video 1 The TSURECOMI Technique
